# Supplementary material for: Phenotypic decanalization driven by social determinants could explain variance patterns for glycemia in adult urban Argentinian population
Source: Sci Rep. 2022 Jun 27;12:10865. doi: 10.1038/s41598-022-15041-9 (PMC9237041; doi:10.1038/s41598-022-15041-9)
Supplement: Supplementary file 1 — Supplementary Legends. [file 41598_2022_15041_MOESM1_ESM.docx]

Additional File 1: Data from the National Health Survey for all individuals retained.
