# Supplementary material for: Phenotypic decanalization driven by social determinants could explain variance patterns for glycemia in adult urban Argentinian population
Source: Sci Rep. 2022 Jun 27;12:10865. doi: 10.1038/s41598-022-15041-9 (PMC9237041; doi:10.1038/s41598-022-15041-9)
Supplement: Supplementary file 3 — Supplementary Information 2. [file 41598_2022_15041_MOESM3_ESM.doc]

Additional File 2. Summary for all covariates used as explanatory variables in the analyses for all individuals of urban areas in which glycemia was (n=4477) or was not (n=6461) measured. Note: this table contains information from all individuals, including incomplete cases removed for the final analysis (see Table 1).

|  | With glycemia test | No glycemia test |
| --- | --- | --- |
| Mean ± SD age (years) | 50.3 ± 16.2 | 50.2 ± 16.4 |
| % women | 40.9 | 43.7 |
| Mean ± SD income (AR$) | 22999.2 ± 17991.3 | 24301.3 ± 18473.7 |
| % high working hours | 15.5 | 16.8 |
| % low working hours | 22.9 | 21.3 |
| % unemployed | 38.9 | 36.7 |
| % lower educ attainment | 8.7 | 6.4 |
| % higher educ attainment | 33.9 | 31.1 |
| % high physical activity | 16.1 | 16.6 |
| % low physical activity | 47.1 | 45.8 |
| Mean ± SD sitting minutes | 259.9 ± 173.4 | 275.0 ± 174.9 |
| Mean ± SD daily fruit (portions) | 2.1 ± 1.7 | 2.2 ± 1.7 |
| % high alcohol consumption | 3.7 | 3.1 |
| % low alcohol consumption | 82.8 | 84.9 |
| % high salt consumption | 10.1 | 11.8 |
| % no salt consumption | 21.1 | 22.8 |
