# Supplementary figures and images for: Phenotypic decanalization driven by social determinants could explain variance patterns for glycemia in adult urban Argentinian population
Source: Sci Rep. 2022 Jun 27;12:10865. doi: 10.1038/s41598-022-15041-9 (PMC9237041; doi:10.1038/s41598-022-15041-9)

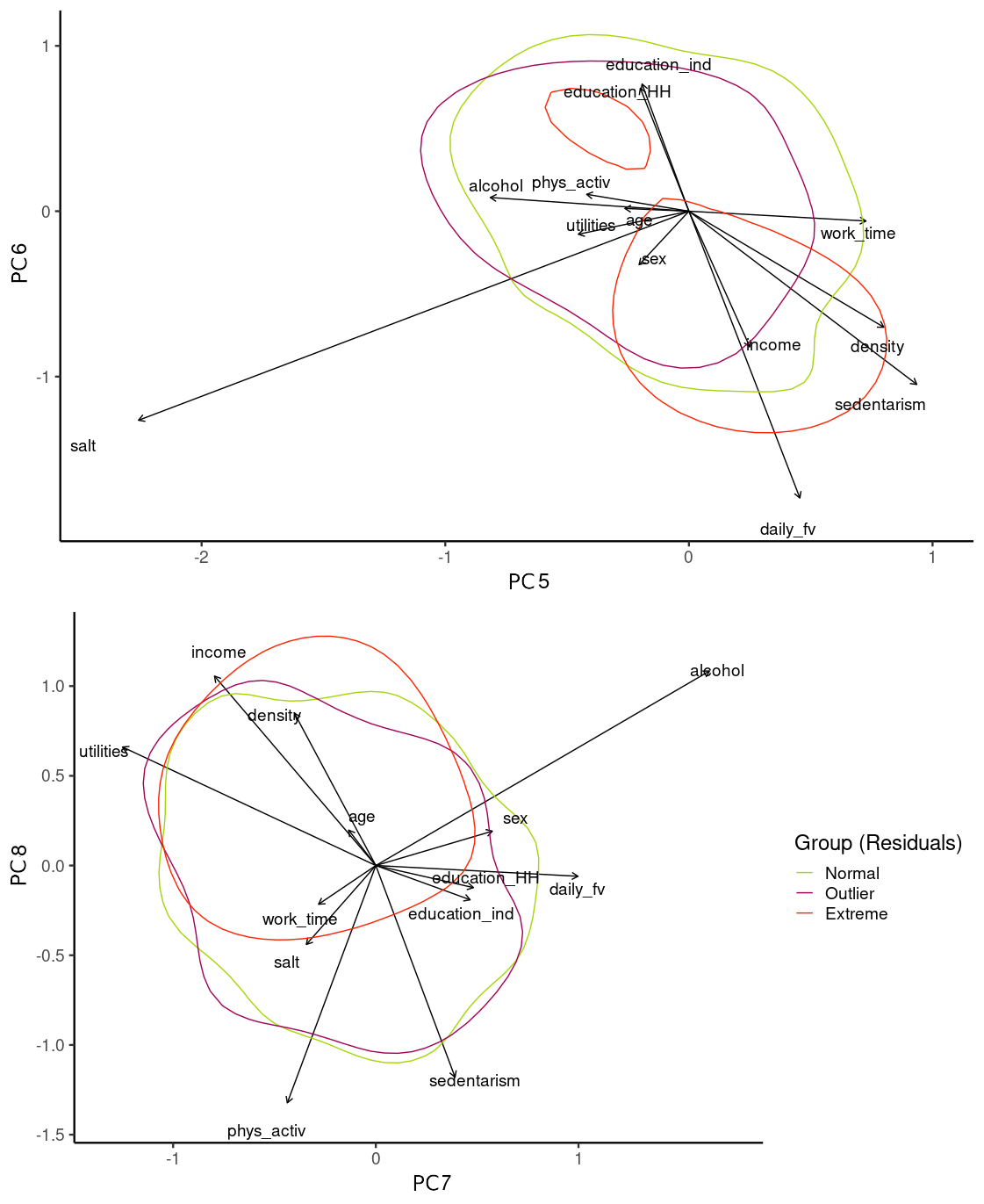

Supplement: Supplementary file 6 — Supplementary Information 5. [file 41598_2022_15041_MOESM6_ESM.png]

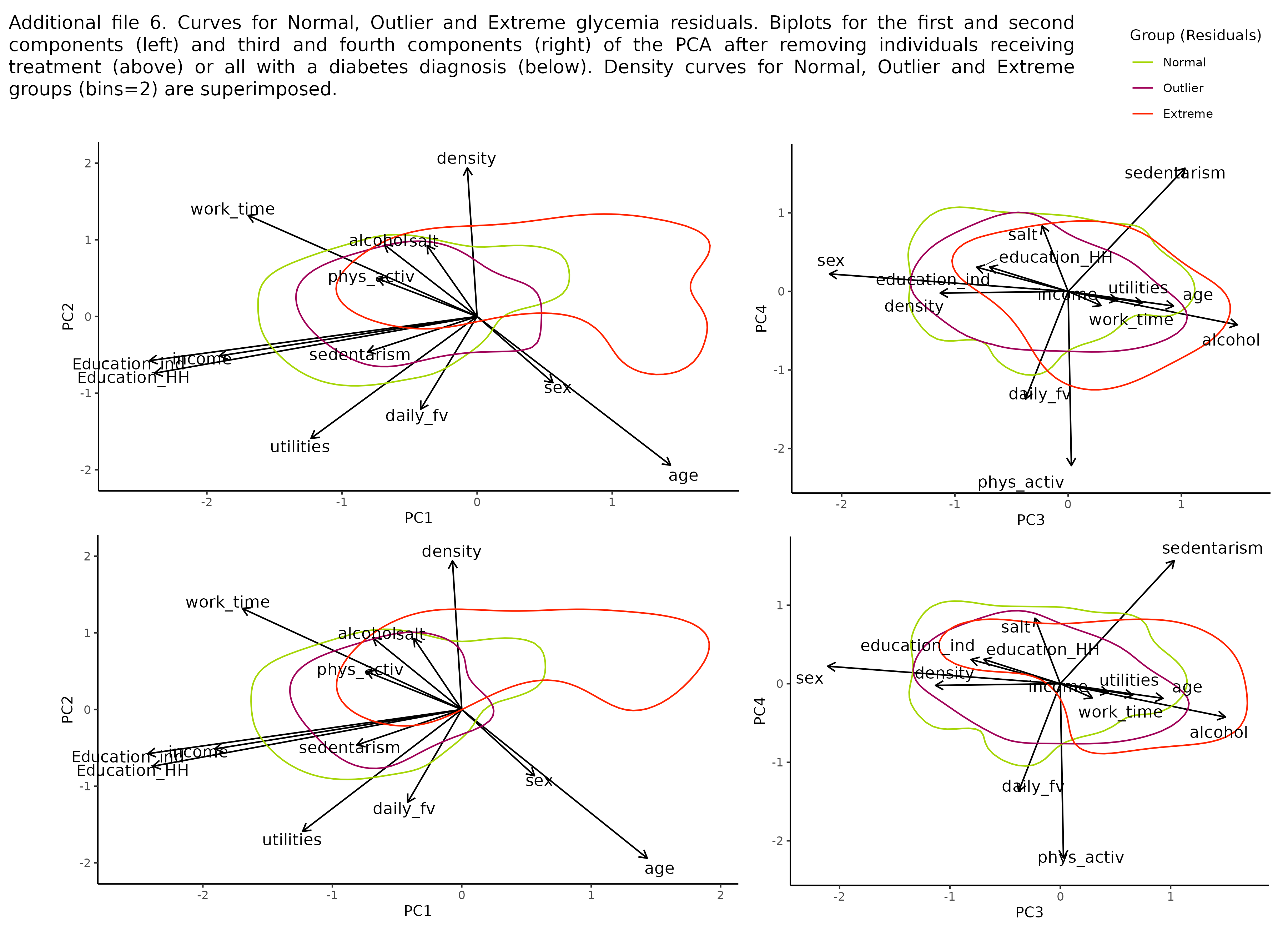

Supplement: Supplementary file 7 — Supplementary Information 6. [file 41598_2022_15041_MOESM7_ESM.png]

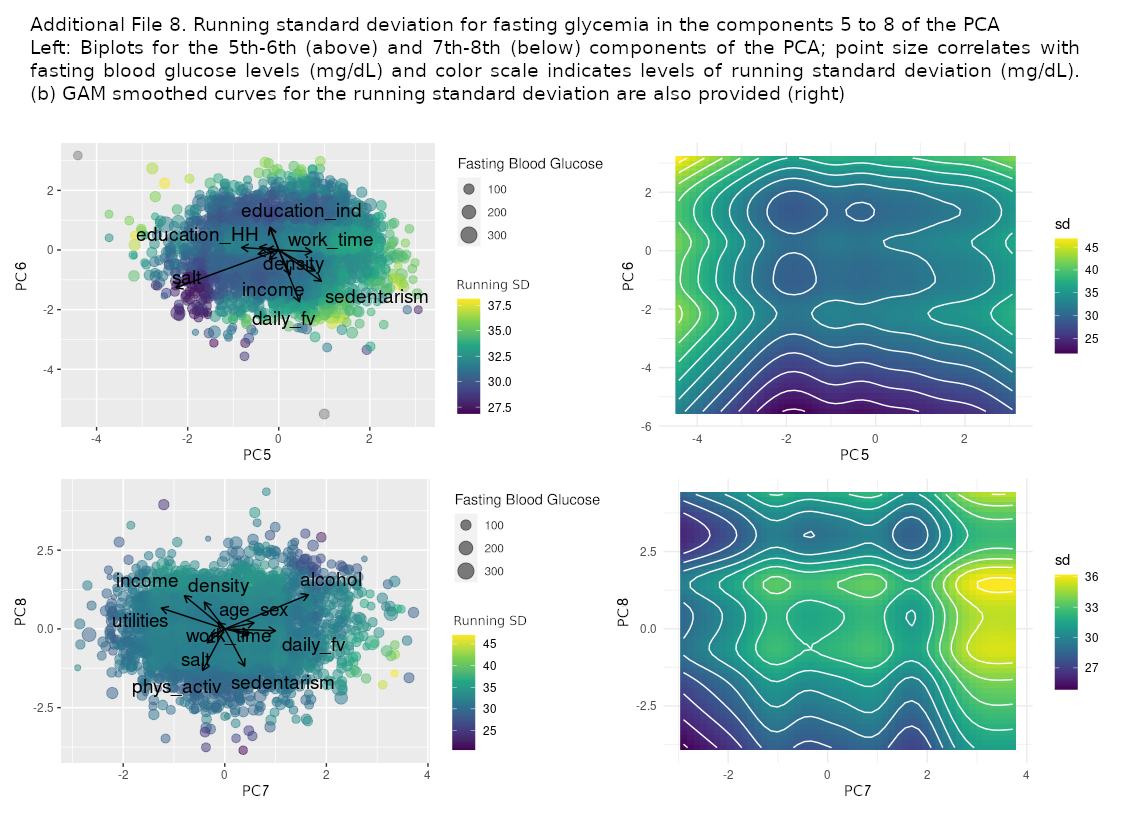

Supplement: Supplementary file 9 — Supplementary Information 8. [file 41598_2022_15041_MOESM9_ESM.png]
