# Supplementary material for: Phenotypic decanalization driven by social determinants could explain variance patterns for glycemia in adult urban Argentinian population
Source: Sci Rep. 2022 Jun 27;12:10865. doi: 10.1038/s41598-022-15041-9 (PMC9237041; doi:10.1038/s41598-022-15041-9)
Supplement: Supplementary file 8 — Supplementary Information 7. [file 41598_2022_15041_MOESM8_ESM.docx]

Additional File 7. Results of the pairwise comparisons for the PERMANOVA test

|  | F | Adjusted p-value |
| --- | --- | --- |
| Normal - Extreme | 7.22 | 0.003 |
| Normal - Outlier | 4.43 | 0.003 |
| Extreme - Outlier | 3.38 | 0.009 |
