# Supplementary material for: Phenotypic decanalization driven by social determinants could explain variance patterns for glycemia in adult urban Argentinian population
Source: Sci Rep. 2022 Jun 27;12:10865. doi: 10.1038/s41598-022-15041-9 (PMC9237041; doi:10.1038/s41598-022-15041-9)
Supplement: Supplementary file 10 — Supplementary Information 9. [file 41598_2022_15041_MOESM10_ESM.doc]

**Additional methodological note**

Explanatory variables considered:

education_ind and education_HH (educational attainment for the interviewed individual and head of the household):

1 = elementary school not completed

2 = completed elementary school, high school not completed

3 = completed high school

density (ratio of inhabitants / number of rooms on the household)

utilities (a joint variable combining the information for water, gas and sewage system availability, rescaled between 0 and 1)

work_time (paid working time):

0 = unemployed or retired interviewees, and interviewees doing only unpaid work

1 = time < 35 weekly hours

2 = 35 < time < 45 weekly hours

3 = time > 45 weekly hours

daily_fv (average number of daily fruits and vegetables consumed)

alcohol (levels of alcohol consumption):

0 = no problematic consumption

1 = problematic regular consumption or problematic episodic consumption

2 = problematic regular consumption and problematic episodic consumption

salt (levels of salt intake):

0 = does not use salt, to 3 = always adds salt to meals

phys_activ (levels of physical activity as recorded in the survey):

1 = low

2 = intermediate

3 = high

sedentarism (daily minutes spent sitting)

Categorized fasting glycemia levels (in mg/dL)

1 (glycemia<70), 2 (70<=glycemia<110), 3 (110<=glycemia<140), 4 (140<=glycemia<200), 5 (200<=glycemia<240) and 6 (glycemia>=240)

Statistical analyses:

Random Forest works by repeatedly subsampling from the data, constructing a decision tree for each subsample (in the case of regression, the response variable is discretized) and then averaging or combining the resulting trees.

Gradient Boosting Machine also works with trees, by producing an ensemble of “weak” ones into a “strong” classifier, where each decision tree has a different influence according to performance.

Support Vector Machine works by finding a hyperplane defined by the optimal parameters to explain the response variable (i.e. minimizing the distance to the maximum number of points).

Categorical PCA: combines linear multivariate analysis with optimal transformation of the categorical variables using alternating least squares.
